# *Sanghuangporus toxicodendri* sp. nov. (Hymenochaetales, Basidiomycota) from China

**DOI:** 10.3897/mycokeys.57.36376

**Published:** 2019-08-22

**Authors:** Sheng-Hua Wu, Chiung-Chih Chang, Chia-Ling Wei, Guo-Zheng Jiang, Bao-Kai Cui

**Affiliations:** 1 Department of Biology, National Museum of Natural Science, Taichung 40419, Taiwan National Museum of Natural Science Taichung Taiwan; 2 Paoshantang Medicinal Herbs Development Co., Ltd, Xizang 850100,China Paoshantan Medicinal Herbs Development Co. Xizang China; 3 Institute of Microbiology, Beijing Forestry University, Beijing 100083, China Beijing Forestry University Beijing China

**Keywords:** *
Inonotus
*, taxonomy, *
Tropicoporus
*, wood-decaying fungi

## Abstract

*Sanghuangporus toxicodendri* (Hymenochaetales) is described as new based on collections made from Shennongjia Forestry District, Hubei Province, China. All studied basidiocarps grew on living trunks of *Toxicodendron* sp. This new species is characterized by having perennial, effused-reflexed to pileate basidiocarps; pore surface brownish yellow or yellowish brown, pores 7–9 per mm; context 1–5 mm thick or almost invisible; setae ventricose, dark brown, 26–42 × 7–10 μm; basidia 4-sterigmate or occasionally 2-sterigmate; basidiospores broadly ellipsoid, smooth, brownish yellow, slightly thick-walled, mostly 3.5–4 × 2.8–3 μm. Maximum likelihood and Bayesian inference phylogenies inferred from internal transcribed spacer (ITS) region of rDNA indicated that *Sanghuangporus* spp. formed a monophyletic clade and resolved as a sister to *Tropicoporus* spp., and six strains of *S. toxicodendri* formed a monophyletic group which is sister to *S. quercicola*. An identification key to known species of *Sanghuangporus* is provided.

## Introduction

*Sanghuangporus* Sheng H. Wu et al. and *Tropicoporus* L.W. Zhou et al. were recently segregated from the broad generic concept of *Inonotus* P. Karst (Zhou et al. 2016). The former two genera differ from *Inonotus* s. str. chiefly in having dimitic hyphal system. *Sanghuangporus* is characterized by perennial and effused-reflexed to pileate basidiomata, occurring in a variety of climate environment, whereas *Tropicoporus* is distinguished by annual to perennial basidiomata, and a tropical distribution (Zhou et al. 2016). Zhu et al. (2019) showed the molecular phylogeny strongly supports the monophyly of *Sanghuangporus* spp.; they also indicated that the maximum crown age of *Sanghuangporus* is approximately 30.85 million years, and East Asia is the likely ancestral area. *Sanghuangporus* spp. usually have host-specificity relationships with their host trees. *Sanghuangporus* accommodates some important medicinal fungal species generally are called “Sanghuang” (means yellow organism grows on *Morus*) in China and Korea, and “Meshimakobu” in Japan. *Sanghuangporus sanghuang* (Sheng H. Wu et al.) Sheng H. Wu et al., the generic type, was detected by Wu et al. (2012) as the genuine Sanghuang species growing exclusively on *Morus* in the wild. Before this study, 13 species of *Sanghuangporus* were known (Ghobad-Nejhad 2015; Tomsovsky 2015; Zhou et al. 2016; Zhu et al. 2017). In this study, we present a new species of *Sanghuangporus* sp. growing on *Toxicodendron* sp. collected from Shennongjia Forestry District, Hubei Province of China.

## Materials and methods

### Morphological studies

All studied specimens are deposited in the herbarium of National Museum of Natural Science, ROC (TNM). The description is based on dried basidiocarps. Freehand and thin sections of fruiting bodies were prepared in three media for microscopic studies: 5% (w/v) potassium hydroxide (KOH) with 1% (w/v) phloxine was used for observation and measurement of microscopic characters; Melzer’s reagent was applied to check amyloidity and dextrinoidity; Cotton blue was used to test cyanophily. The abbreviations in the text were used as followed: L = mean spore length (arithmetical average for all spores), W = mean spore width (arithmetical average for all spores), *n* = total number of spores measured from a specimen, Q = variation in the L/W ratio between the studied specimens. When presenting the variation in the dimensions of spores, 5% of the measurements were rejected from each edge of the range and were given in parentheses.

### DNA extraction and sequencing

Genomic DNA were extracted from dried samples with the Plant Genomic DNA Extraction Miniprep System (Viogene-Biotek Corp., New Taipei, Taiwan) following the manufacturer’s protocol. Nuclear ribosomal internal transcribed spacer (ITS) region was amplified with primer pair ITS1/ITS4 (White et al. 1990). The PCR protocols for ITS regions were as follows: initial denaturation at 95 °C for 5 min, followed by 40 cycles at 94 °C for 45 s, 53 °C for 45 s and 72 °C for 45 s, and a final extension of 72 °C for 10 min. PCR products were purified and sequenced by the MB Mission Biotech Company (Taipei, Taiwan). Newly obtained sequences were assembled and manually adjusted when necessary using BioEdit (Hall 1999). The sequences were then submitted to Genbank.

### Alignment and phylogenetic analyses

Zhu et al. (2017) conducted ITS-based phylogenetic analysis for all previously known 13 species of *Sanghuangporus*. The ingroup strains of the *Sanghuangporus* spp. and *Tropicoporus* spp. employed in their analysis were basically adopted in the present analysis. We added newly generated sequences of six strains of the new species (Table 1). *Inonotus rickii* (Pat.) D.A. Reid, the outgroup in Zhu et al.’s analysis was not adopted, as this root failed to separate all *Sanghuangporus* spp. from the *Tropicoporus* spp. We consulted the study of Zhou et al. (2016) and chose *Inocutis tamaricis* (Pat.) Fiasson & Niemelä as the outgroup, which was successful in constructing the tree with a satisfactory result. The dataset was aligned using MAFFT 7 with Q-INS-i strategy. The aligned sequences were manually adjusted in BioEdit (Hall 1999) when necessary. Parsimony informative sites were calculated using MEGA 7 (Kumar et al. 2016). Phylogenetic trees were inferred from Bayesian inference (BI) and Maximum Likelihood (ML) methods using MrBayes v. 3.2.6. (Ronquist et al. 2012) at the CIPRES Science Gateway (http://www.phylo.org/) and PhyML 3.0 (Guindon et al. 2010), respectively. The best fit model for both algorithms was estimated by jModelTest2 (Darriba et al. 2012) using the Bayesian information criterion (BIC). For ML analysis, bootstrap (BS) values were calculated after running 1000 replicates. The BI analysis was conducted with 10 million generations initiated from random starting trees. Trees were sampled every 1000 generations, and the first 2500 trees were discards as burn-in. The Posterior Probability (PP) values were calculated from the remaining trees. Only the phylogram inferred from ML analysis was shown because both BI and ML analyses yield similar topologies. The statistical supports were shown on nodes of the ML tree when BS ≥ 70 and PP ≥ 0.7. The final phylogenetic trees and alignment were submitted to TreeBASE (submission number 24234; http://www.treebase.org).

**Table 1.** List of species, specimens and ITS sequences used in this study. Sequences generated in this study are shown in boldface type.

Species name	Specimen or strain no.	Accession no.
*Sanghuangporus alpinus*	Cui9646	JQ860313
	Cui9658	JQ860310
	Cui9666	JQ860311
*Sanghuangporus baumii*	Cui11903	KY328305
	Dai3694	JN642569
	Dai3684	JN642568
*Sanghuangporus ligneus*	Ghobad-Nejhad 1157	KR073082
	Ghobad-Nejhad 1152	KR073081
*Sanghuangporus lonicericola*	Dai8376	JQ860308
	MG281	KU213574
	TAA55428	JN642575
*Sanghuangporus microcystideus*	AM19	JF895465
	AM-08	JF895464
*Sanghuangporus pilatii*	BRNM 771989	KT428764
*Sanghuangporus quercicola*	Li445	KY328311
	Li1149	KY328312
*Sanghuangporus sanghuang*	BZ-C	JN642587
	Dai12723	JQ860316
	Wu0903-1	JN794061
***Sanghuangporus toxicodendri***	**Wu 1805-2**	**MK400422**
	**Wu 1805-3**	**MK400423**
	**Wu 1805-5**	**MK400424**
	**Wu 1807-2**	**MK729538**
	**Wu 1807-3**	**MK729540**
	**Wu 1807-4**	**MK729539**
*Sanghuangporus vaninii*	Dai3624	JN642590
	SFC 20001106-7	AF534070
	SFCC 10209	AY558628
*Sanghuangporus weigelae*	Cui6012	JQ860319
	WD-1667	JN642594
	Dai11694	JQ860315
*Sanghuangporus weirianus*	CBS_618.89	AY558654
*Sanghuangporus zonatus*	Cui6631	JQ860305
	Dai10841	JQ860306
*Tropicoporus cubensis*	MUCL47079	JQ860325
*Tropicoporus dependens*	JV 1207/3.4-J	KC778779
*Tropicoporus dependens*	JV 0409/20-J	KC778778
*Tropicoporus guanacastensis*	O19228	KP030794
*Tropicoporus linteus*	JV0904/64	JQ860322
*Tropicoporus pseudolinteus*	JV 0312/22.10-J	KC778780
	JV0402/35-K	KC778781
*Tropicoporus sideroxylicola*	JV 1207/4.3-J	KC778783
	JV 0409/30-J	KC778782
*Tropicoporus tropicalis*	CBS-617.89	AF534077
*Inonotus compositus*	Wang 552	KP030781
*Inonotus hispidus*	PST4	EU918125
*Inocutis tamaricis*	CBS 384.72	AY558604

## Results

### Phylogeny results

**Figure 1.**
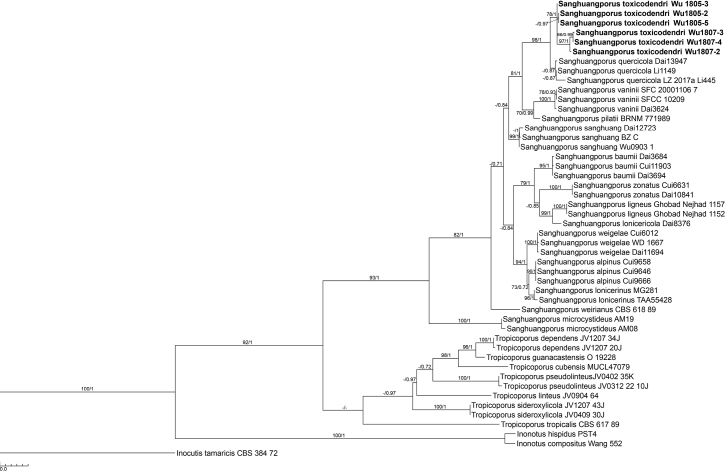
The phylogenetic tree inferred from maximum likelihood and Bayesian analyses of the ITS dataset of *Sanghuangporus toxicodendri* and related species. Statistic supports are shown on internodes with bootstrap values ≥70% and posterior probabilities ≥0.7. The presented new species are shown in boldface type.

The ITS dataset consisted of 48 taxa and 1117 sites including gaps, of which 306 sites were parsimony informative. The HKY+G was selected as the best fit model for both the ML and BI analyses. The BI analysis was terminated when the average standard deviation of split frequencies fell to 0.009547. The ML tree shows that *Sanghuangporus* spp. formed a monophyletic clade (BS = 93%, PP = 1) and resolved as a sister to *Tropicoporus* spp. (BS = 92%, PP = 1) (Fig. 1). Six strains of *Sanghuangporus toxicodendri* formed a monophyletic group with statistical supports (BS = 78%, PP = 1), which was sister to *S. quercicola* L. Zhu & B.K. Cui with significant support (BS = 98%, PP = 1) (Fig. 1).

### Taxonomy

#### Sanghuangporus toxicodendri

Taxon classificationFungiHymenochaetalesHymenochaetaceae

Sheng H. Wu, B.K. Cui & Guo Z. Jiang sp. nov.

09D76A5B989B5463912F32BDC0DE8C83

830791

Figures 2 , 3

##### Type.

CHINA. Hubei Province: Shennongjia Forestry District, Songbai Town, 1200 m, on living *Toxicodendron* sp. trunk, May 2018, *Wu 1805-3* (holotype, TNM F0032663).

##### Etymology.

The epithet refers to the host genus.

##### Description.

Basidiocarps perennial, effused-reflexed to pileate, applanate, semicircular, adaxially slightly concave, woody hard. Pilei projecting 4–6 cm, up to 18 cm wide and up to 6 cm thick at base. Pileal surface grayish black to blackish brown, glabrous, occasionally cracked, concentrically zonate and sulcate; margin generally obtuse, concolorous or brownish yellow. Pore surface brownish yellow, yellowish brown, brownish or rusty brown, somewhat glancing, darkening in KOH; pores 7–9 per mm, circular. Context homogeneous, 1–5 mm thick or almost invisible, brownish yellow or brownish, with blackish crust at pileus parts. Tubes concolorous with pore surface, 1–5 cm thick, usually with several growth layers.

Hyphal system dimitic in both context and trama, generative hyphae simple-septate; tissue darkened in KOH. Context generative hyphae yellowish, brownish yellow or yellowish brown, moderately ramified, 2–3 μm diam., slightly thick-walled or with walls up to 1 μm thick; skeletal hyphae yellowish brown to brownish, fairly straight, rarely ramified, 2–4 μm diam., with 0.5–1.3 μm thick walls or subsolid. Tube generative hyphae yellowish brown to yellowish, moderately ramified, 2–3 μm diam., slightly thick-walled or with walls up to 1 μm thick; skeletal hyphae yellowish brown to brownish, fairly straight, rarely ramified, 2–4 μm diam., with 0.8–1.3 μm thick walls or subsolid. Hymenial setae ventricose, dark brown, 26–42 × 7–10 μm. Cystidioles with tapering or abruptly narrow apices, colorless, thin-walled, 10–20 × 3–3.5 μm. Basidia clavate, 10–12 × 4–4.5 μm, thin-walled, 4-sterigmate or occasionally 2-sterigmate; sterigmata up to 6 μm long. Basidiospores broadly ellipsoid, smooth, brownish yellow, slightly thick-walled, inamyloid, non-dextrinoid, somewhat cyanophilous, (3.2–)3.5–4 × (2.7–)2.8–3(–3.2) μm, L = 3.72±0.21 μm, W = 2.94±0.11 μm, Q = 1.27 (*n* = 30, holotype: *Wu 1805-3*).

##### Ecology and distribution.

On trunk of *Toxicodendron* sp. Hitherto only known from Shennongjia Forestry District, Hubei province, China.

##### Additional specimens examined (paratypes).

CHINA. Hubei Province: Shennongjia Forestry District, Songbai Town, 1200 m, on living *Toxicodendron* sp. trunk, May 2018, *Wu 1805-1* (TNM F0032661), *Wu 1805-2* (TNM F0032662), *Wu 1805-4* (TNM F0032664), *Wu 1805-5* (TNM F0032665); July 2018, *Wu 1807-2* (TNM F0032666), *Wu 1807-3* (TNM F0032667), *Wu 1807-4* (TNM F0032668).

**Figure 2.**
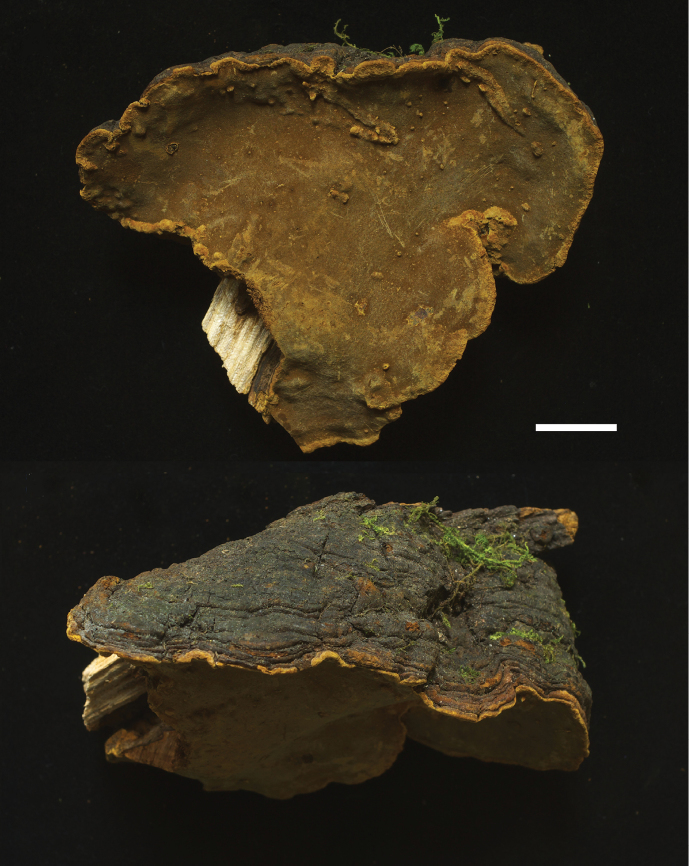
Basidiocarp. *Sanghuangporus toxicodendri* (holotype, *Wu 1805-3*).

**Figure 3.**
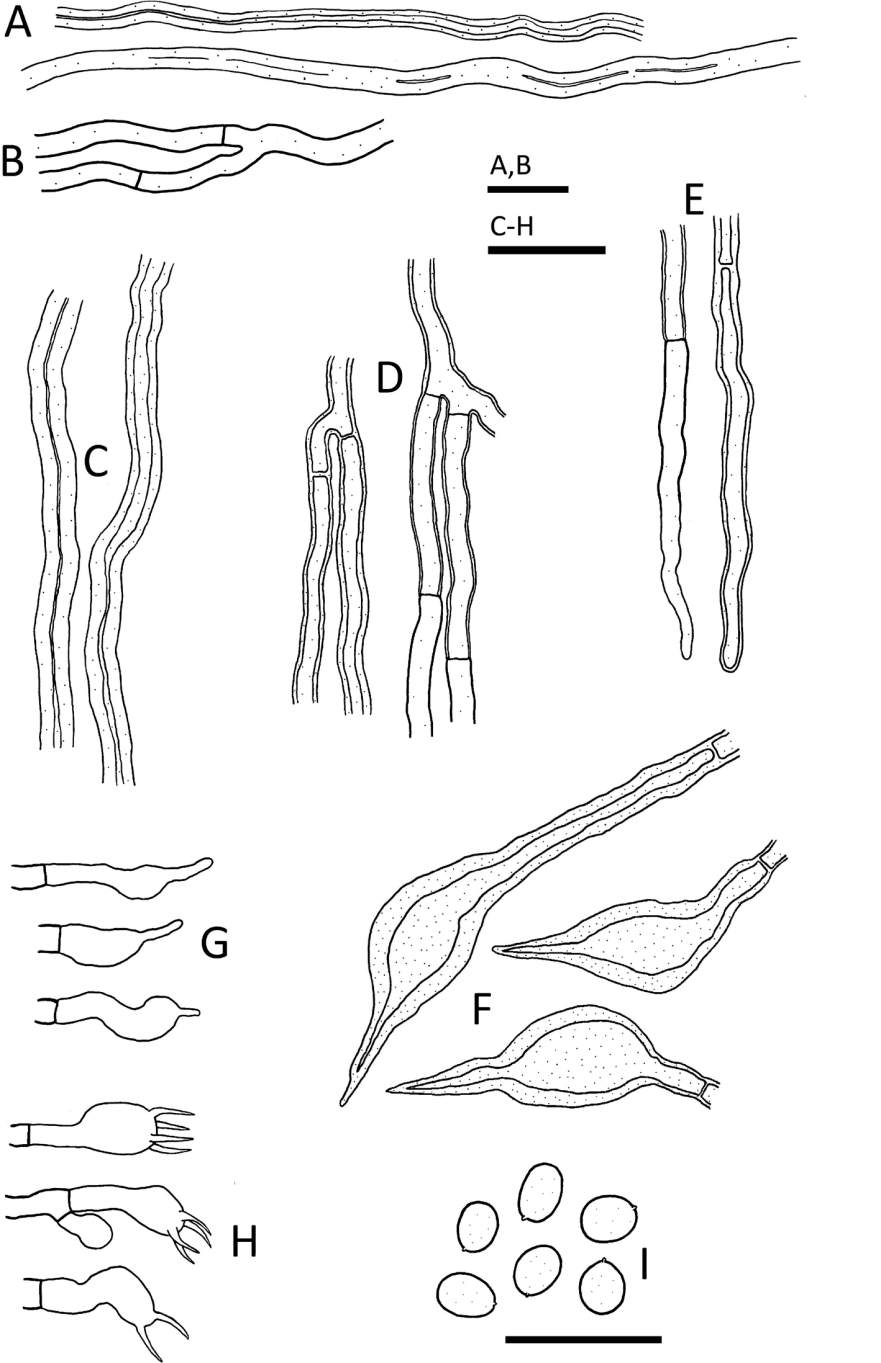
*Sanghuangporus toxicodendri* (holotype, *Wu 1805-3*) **A** skeletal hyphae from context **B** generative hyphae from context **C** skeletal hyphae from trama **D** generative hyphae from trama **E** generative hyphae from dissepiments **F** setae **G** cystidioles **H** basidia **I** basidiospores. Scale bars: 10 μm.

## Discussion

Zhu et al.’s (2019) phylogenetic study showed the monophyly of the genus *Sanghuangporus* spp., and the result coincides with the present study (Fig. 1). The genus *Sanghuangporus* comprises 14 species (Ghobad-Nejhad 2015; Tomsovsky 2015; Zhou et al. 2016; Zhu et al. 2017), after including *S. toxicodendri* presented here. It is not easy to identify some species of *Sanghuangporus* spp., as there are not that many good morphological characteristics to separate them. Distribution, climatic adaptation, host preference, and DNA sequences are important for species recognition, apart from morphological study.

The present phylogenetic study indicated that *S. toxicodendri* is sister to *S. quercicola* with significant support (Fig. 1). Both species are distributed in central China; the former grows on *Toxicodendron*, while the latter occurs on *Quercus*. However, two morphological features can separate these species. The yellow or brownish-yellow wide marginal zone on the pileus surface of *S. quercicola* (Zhu et al. 2017: figs A, B) is lacking in *S. toxicodendri*. Secondly, the basidiospores of *S. toxicodendri* are mostly longer than 2.8 μm, but are generally shorter than 2.8 μm in *S. quercicola*.

*Sanghuangporus lonicericola* (Parmasto) L.W. Zhou & Y.C. Dai, *S. quercicola*, *S. sanghuang*, *S. toxicodendri*, *S. vaninii* (Ljub.) L.W. Zhou & Y.C. Dai, and *S. zonatus* (Y.C. Dai & X.M. Tian) L.W. Zhou & Y.C. Dai have comparatively smaller pores (>6 per mm) than other species. *Sanghuangporus lonicericola* is distributed in northeast China and the Russian Far-East, growing exclusively on *Lonicera*; moreover, it has smaller setae (12–22 × 4–8 μm; Dai 2010) than *S. toxicodendri*. *Sanghuangporus sanghuang* grows only on *Morus* and has distinctly larger basidiospores (4–4.9 × 3.1–3.9 μm; Wu et al. 2012) than *S. toxicodendri*. *Sanghuangporus vaninii* grows on *Populus* and also resembles *S. quercicola* in having a wide marginal yellow zone on pileus surface, but it has larger basidiospores (3.8–4.4 × 2.8–3.7 μm; Dai 2010) than *S. toxicodendri*. *Sanghuangporus zonatus* is a tropical species distributed in southern China and differs from *S. toxicodendri* in having thicker context and shorter setae (Tian et al. 2013).

Several *Sanghuangporus* spp. are used for medicinal application in China, Korea, Japan, and South Asian countries. Wu et al. (2012) indicated that *S. sanghuang*, the only *Sanghuangporus* sp. growing on *Morus* in the wild, is the genuine Sanghuang species. Comparing health-care effectiveness among the so-called Sanghuang species, Lin et al. (2017) proved that *S. sanghuang* has better medicinal properties than two other commercial species: *S. baumii* (Pilát) L.W. Zhou & Y.C. Dai and *S. vaninii*. *Sanghuangporus vaninii* grows on *Populus davidiana* in the wild and is widely cultivated in China, Korea, and Japan as a medicinal fungus. *Sanghuangporus baumii*, which grows on *Syringa* in the wild, is also served as medicinal fungus in China. The medicinal properties of many *Sanghuangporus* spp. are not understood. It is noted that *S. toxicodendri* and the recently described *S. quercicola* are closely related to the medicinal species *S. sanghuang* and *S. vaninii* (Zhu et al. 2019; this study, Fig. 1). The medicinal properties of these two species are worth studying.

### Key to the accepted species of *Sanghuangporus*

**Table:** 

1	Pores 3–5 per mm	**2**
–	Pores > 5 per mm	**3**
2	Basidiospores 3.5–4.5 × 3–3.5 μm; distribution in Central Asia	***S. lonicerinus***
–	Basidiospores 4–4.8 × 3–3.8 μm; distribution in Europe	***S. pilatii***
3	Pores 7–10 per mm	**4**
–	Pores 5–8 per mm	**6**
4	Brownish yellow pileus surface marginal zone present; restricted to *Quercus*	***S. quercicola***
–	Brownish yellow pileus surface marginal zone not present; not on *Quercus*	**5**
5	Setae >25 μm long; restricted to *Toxicodendron*	***S. toxicodendri***
–	Setae <25 μm long; restricted to *Lonicera*	***S. lonicericola***
6	Context very thin, <3 mm	**7**
–	Context very thick, >10 mm	**8**
7	Context duplex; distribution in the warm temperate zones	***S. weigelae***
–	Context homogeneous; distribution in alpinus zones	***S. alpinus***
8	Setae mostly <20 μm long	**9**
–	Setae mostly >20 μm long	**12**
9	Basidiomata with a sharp margin	***S. zonatus***
–	Basidiomata with an obtuse margin	**10**
10	Basidiospores basically subglobose; distribution in Africa	***S. microcystideus***
–	Basidiospores broadly ellipsoid; distribution in Asia	**11**
11	Dissepiments distinctly thick; distribution in western Asia	***S. ligneus***
–	Dissepiments distinctly thin to slightly thick (<¼ diameter of pores); distribution in eastern Asia	***S. baumii***
12	Basidiospores basically subglobose; restricted to *Juglans*	***S. weirianus***
–	Basidiospores broadly ellipsoid; restricted to *Morus* or *Populus*	**13**
13	Basidiospores 3.8–4.4 × 2.8–3.7 μm; restricted to *Populus*	***S. vaninii***
–	Basidiospores 4–4.9 × 3.1–3.9 μm; restricted to *Morus*	***S. sanghuang***

## Supplementary Material

XML Treatment for Sanghuangporus toxicodendri
